# Needle Found in the Heart after Death

**Published:** 1849-03

**Authors:** 


*Needle found in the Heart after death.* Reported by John Neill, M. D., Demonstrator of Anatomy in the University of Pennsylvania.

Upon the dissection of a black male subject, brought into the anatomical room about the middle of December, my attention was directed, by a student, to a foreign body in the heart. At first, I supposed that it might have been introduced after death, accidentally dropping into the cavity of the pericardium, during the process of stitching after injection; but upon more careful examination of the surface of the heart, no orifice was detected by which it could have entered. I removed the heart and placed it in alcohol, in order to examine it with care.

The pathological condition of the contiguous viscera could not be made out very satisfactorily, on account of the length of the period which had elapsed since death, and from the fact, that an antiseptic injection (chlor. of zinc) had been used, which destroys color and coagulates albumen; there were, however, marks of chronic disease evident, in adhesions of the pleura and serious pericardium; there was also evidence of peritoneal inflammation.

After the heart had been hardened in alcohol, and cleanly washed of clots, I found imbedded in the external wall of the left ventricle, a broken needle, with its point directed forwards towards the apex of the heart; it was much oxidized, and could not be moved from its position, until the cyst containing it was split up. The broken end encroached upon the cavity of the ventricle, being actually contained in one of the columnæ carneæ; the needle was two inches in length, and a line in thickness, belonging to a variety called *worsted needles.*

In the Medical Examiner for May, 1843, Dr. Leaming reports a case of a seamstress, who had accidentally driven a needle, which was sticking in her dress, forcibly into breast, by striking a table. In a month she had pleurisy, and subsequently pericarditis and pneumonia, and at the end of nine months she died. The post-mortem examination revealed lesions, corresponding with the symptoms; the body of the needle was found imbedded partly in the wall of the right ventricle, and partly in the vetricular septum, whilst the point projected for a quarter of inch into the cavity of the left ventricle.

In the summary of the American Medical Journal, a case is copied from the Archives Generales, 1842, in which a soldier introduced two needles into his heart, and was brought screaming into the hospital at St. Petersburgh; he had a hard, quick pulse; anxious countenance; copious perspiration; distressing cough, and tumultuous action of the heart; in nineteen days he died; and upon examination after death, it was discovered that the needles had passed through the heart, and lodged in the lower part of the left lung, where they were found in an abscess. The whole track was easily recognised by the marks of inflammation.

In the Annalist for November, 1847, Dr. Graves records a case of attempted suicide. A man pushed a needle into his heart, expecting instant death, as in the instance of Admiral Villeneure, after the battle of Trafalgar; but being disappointed in the immediate effect, he undertook to cut his throat, which also failed; the vessels having been secured, and the wound dressed by his medical attendant. After reaction had taken place, he had great suffering; every breath being attended with a scream; the physician discovered the puncture made in the skin by the needle, and dissected through the intervening structures, until he "could distinctly see the heart pulsating with the needle in it." "With the aid of a pair of forceps, I extracted the needle, and it was followed with a forcible stream of blood." "He continued to improve up to the sixth day, when he was attacked with pleuritic pains, and inability to swallow; and died on the eighth day after the needle was taken from the heart." *Post-mortem.*—"On opening into the left ventricle, where the needle entered the cavity, there was a small membranous sac, about the size of a pea, formed in the left ventricle, which contained pus."

NOTE.—I learn, through the politeness of Dr. Klapp, physician to the Moyamensing prision, that this man was admitted May 11th, 1847, in rather feeble health; but continued to work for more than a year before complaining of any inconvenience about his chest. When removed to the infirmary, he had severe cough, with some slight constriction in breathing, and occasional palpitation. These symptoms, though never very urgent, continued until his death. Though never delirious, and able to answer questions to the last, he never spoke of having received any injury of the kind, and never manifested any suicidal tendency.—*Medical Examiner.*

